# News and Miscellany

**Published:** 1884-04

**Authors:** 


### NEWS AND MISCELLANY.

Inoculation of Dogs with Syphilis.—Dr. Lentick and Prof. Reinhart, of Odessa, have attempted to inoculate dogs and pigs with syphilis. Although every precaution was used in the experiments, none of the inoculations proved successful; one of the dogs, however, died of septicemia. From their observations they conclude that syphilis cannot be given to animals.—*Weekly Medical Review*.

A Cowman Attacked by Foot-and-Mouth Disease.—A correspondent of the *Live Stock Journal* relates a case of foot-and-mouth disease in the human subject as having recently occurred at Market Drayton. The patient, a cowman, appears to have been inoculated in the mouth with the virus from an animal to which he was administering a drink. The report says: "His mouth has been one mass of sores, which apparently have been continued throughout the whole interior of his body, and now he is breaking out in similar ulcers on the legs and feet." He has been attended by three medical men, and the above statement is made on the authority of one of them. In our leader of September 26th on "The Milk from Cows suffering from Foot-and-mouth Disease" attention was drawn to the fact that several cases of infection from animal to man had been recorded. If the one under consideration is well authenticated—and there seems no reason to doubt this—there is additional evidence of the risks incurred by persons who have charge of diseased cattle. Collective investigation could scarcely fail to give some valuable information both as regards the frequency of communication to man and the variation and course of the symptoms.

Scenes in Pasteur's Workshop.—The following somewhat gruesome description of M. Pasteur's laboratory, taken from a recently published volume entitled "L'Histoire d'un Savant par un Ignorant," gives a very striking picture of the modern physiological workshop: "All the animals in the laboratory, from the little white mice hiding under a bundle of cotton wool to the downwards. Turning the edge towards the ligament I easily made the section, which announced itself by a crackling sound. The external wound was closed by carbolised dressing.

Eight days after the operation the animal went to pasture without a sign of of lameness and the cramp never returned.

*Cravenna Santo, in Il Medico Veterinario, Torino.*

Bacteria in the Horse.—In consideration of the uncertainty and obscurity which reigns in relation to post mortem appearances in some diseases of the horse of the bacteria of the blood which are confounded with those of anthrax, I will relate two clinical cases, one of *Hemorrhagic Typhus*, and the other of *Traumatic Gangrene.*

*First Case.*—The first symptoms which declared itself was epistaxis which was combatted by cold applications, injections of the perchloride of iron and tamponing the nasal cavities but without success.

The hemorrhage continuing and the temperature rising I concluded that I had more than a simple epistaxis to deal with and diagnosing Hemorrhagic Typhus, administered a large dose of quinine. This was of no avail as the animal became worse and died on second day of illness.

Post mortem examination revealed numerous sub-cutaneous ecchimoses and extravasations of dark blood in various parts of the body. Bacteria, resembling those of anthrax were found in the blood upon examination by the microscope. On comparing them with the bacteria taken from the blood of a rabbit suffering from anthrax they were found to differ in appearance, being larger and longer, and this difference proved real on treating them in accordance with Pasteur's method. Furthermore animals inoculated with the blood taken from the cadaver gave no evidence of anthracoid infection.

The second case was observed in a horse in which an amputation of an excresence in the cord of the testicle had been performed. Antiseptic treatment was adopted but the part operated soon became enormously swollen, gangrene set in and the animal died with symptoms of grave septic infection. Bacteria were found in the blood. They were shorter and more controlled than those found in the first horse. Inoculation gave the same results as in the first case.

We must conclude that the bacteria in these two cases are of two particular species. Are they characteristic of a known septicemia? They are unlike those described by Pasteur, Koch, Davaine or Toussant. Are they *post mortem* emigrants from the intestine as observed by Lewes ?

At any rate they are intimately connected with that disease which kills so many horses.

*D. Vigezzi and S. Rivolta in Giornale di Anat, Fisiol and Patol, degli animale, Pisa.*

Cancer in Domesticated Animals.—At present, when the relations between the disease of man and his dependent cousins are being realized and brought to notice, and are influencing many minds to advancement in learning and to the broadening of their field of view, an account of cases such as the following needs no apology for intruding itself on the time of the thinking portion of the medical profession. Their clinical interest is not so certain, but, *a priori*, it may be questioned if the food from animals in whose bodies degraded material processes are going on can be any more edifying than the mental pabulum of the same description is by some held to be. If the existence of a mammary cancer in a woman would, in the judgment of a thoughtful practitioner, be good cause for doubting the advisability, surely the existence of cancerous ulceration in the udder of a cow would be good cause, for the disallowance of the use of her milk for the food of young children.

The investigation whose results this paper offers was undertaken to obtain material for the inflammatory origin of tumors. But time went on its way, the paper was never written, and now it is offered as a contribution to the general stock-in-trade of the pathologist. Its intrinsic interest is not lessened even if it is regarded as a curiosity only; but it also indicates that, besides enteric fever, pneumonitis, and tuberculois, another vice of nutrition is shared by us all.

*Case I.*—A chicken whose humerus had been broken. Three months after, a lump the size of an egg was present. In the centre of this mass the ununited fracture could be perceived. Death followed from exhaustion after some months. The lump was a very finely developed, large, round-celled sarcoma. The cells were in nests proliferating, some multinucleated. There was no proper callous formation, but the ends of the bones were swollen, softened, and covered with the cell-deposit. The lymphatic glands were not inflamed. The tumor weighed 64 grammes.

*Case II.*—An old hen. Feet frost-bitten. A piece of superficial tissue about two centimetres in diameter came away from one foot. The resulting sore was treated with the compound tincture of benzoin and fresh lard, equal parts, made into an ointment. The right foot healed completely; the left nearly so. This hen hatched a brood of chickens in the spring. The next time I noticed her, about midsummer, she was moving in so peculiar a manner that I caught her, made an examination, and found the left sole occupied by an elastic mass of about the size of a pigeon's egg. The leg soon became useless, and the hen died marasmic. On post-mortem, I found the lymphatic glands enlarged, containing multinucleated cells, the stroma proliferating *pari passu*. The extremity of the tarso-metatarsal bone was a mass of encephaloid disease.

*Case III.*—A capon. I removed the glands by the usual operation. The capon died, at the end of three months, of acute peritonitis. He was as large as a turkey. The post-mortem showed that the peritonitis was caused by the bursting of a cyst containing some irritant fluid, which cyst formed part of a tumor as large as an egg, occupying the place of the right testicle. This tumor was an adenoid sarcoma. The size of the tumor was remarkable in view of the copon's development.

*Case IV.*—A fine setter. Wounded in the mouth by the discharge of a gun. The canine tooth on the lower jaw, right side, was so loosened as to be disengaged by very gentle handling. Ulceration of the socket followed, and after a considerable period a tumor grew, whose size made the dog so miserable that he was allowed to die. The microscope sections thence obtained might have served as originals for the drawings of osteo-sarcomas shown in the books.

The next four cases of which I have memoranda were in horses and mules, the result of injuries from the abuse of bits. In one of them there was an exostosis, or else a sub-mucous thickening of the roof of the mouth. The animals were otherwise in good condition.

My next was a large, mixed spindle and round-celled sarcoma from the lower lip of a mule, caused by the irritation of the bit.

*Case X.*—A papilloma, irritated by the rubbing of the bridle behind the ear of a horse, became ulcerated. I removed it and obtained some excellent sections of a connective-tissue tumor, with nests of pseudo-epithelial cells.

*Case XI.*—Last spring, while visiting a patient, I learned that one of his cows was "snake-bit." Investigation showed an ulcerated mass on the udder that macro and microscopically answered to a nondulated mammary cancer.

A case of a gastric tumor in a pig was offered at the Chicago meeting of the American Society of Microscopists.

Epithelial tumors at the point of the shoulders in abused horses are not very scarce.

Engorgement of the lymphatics of the base of the tongue following wounds that healed by leaving an indurated cicatrix have passed under my notice; also one case of a lump, of the size of a fist, that formed about the parts pressed against by a ring in a bull's nose. There was nothing destructive about this tumor, but its evident inflammatory character renders it worth mentioning.

Villous tumors of the intestine are not infrequently found in hogs.

The fact that lymphatics may swell and become indurated in pigs as the result of dysentery, and then present very suspicious appearance under the microscope, opens a field that needs further investigation. From my own study in this direction, I am inclined to the conclusion that, as all nutritive processes as a rule are more rapid, and healing processes are more complete, in the lower animals than in mankind, interruptions and maldirection of these processes are productive of less diastrous results. Still there is evidence to be found among them, and among them alone, that makes the *modus vivendi* of the tumor brotherhood much clearer than in man. In dumb animals only can we find very young growths and remove them at our will, or remove all the neighboring tissue and appreciate its relations.—*Dr. W. H. Birchmore in the New York Medical Journal.*

Treatment of Glanders and Farcy by the Dosimetric System of Therapeutics.—In the January number of the *Journal of Medicine and Dosimetric Therapeutics*, several cases are recorded of the cure of glanders by the employment of remedies prepared and exhibited according to the above-named system.

We may state that dosimetry is the method of cure which consists in the use of alkaloids, or active principles of various drugs in a pure form, and in extremely small doses in the form of granules. Dosimetry is not at all allied to homœopathy, as it does not proceed on the principle which is expressed in the words *similia similibus curantur;* but rather on the principle of alopathy, excepting that all the ordinary drugs are rejected in favour of their active constitutents only.

Some of the most potent alkaloids are prepared in granules containing half a milligramme of active substance, and five of these granules are a dose for a horse. Among these preparations we observe arseniate of strychnine, atropine, veratrine, etc.; other granules contain one milligramme of active substance, such as arsenious acid. The strongest granules in the list of medicine contain one centigramme, and in this list are salicylic acid and tannin. Some idea of the smallness of the dosimetric dose may be gained from a comparison of the allopathic doses of the same agents. A drachm of tannin might be given, and repeated at short intervals, in cases of internal haemorrhage in the horse or ox; and thirty grains of salicylic acid three times a day would not be an excessive dose—instead of five granules, containing a centigramme in each, of the same agents, the whole dose amounting to something like half a grain per day. Much less doses are given when the most active alkaloids are used; still the doses do not approach in minuteness the dilutions of the homœopathic therapeutist.

The cases of glanders which were successfully treated by dosimetry were recorded by M. Camoin, an eminent veterinary surgeon in Boufarik, Algeria, and M. Baumann, formerly in the Belgian army and now practising in the Ardennes, who has forwarded a report on the subject to the Minister of Agriculture. Three cases of glanders are recorded by the first named veterinarian, and two by the latter.

In the first cases a farmer had remarked that a bay mare, eight years of age, presented symptoms of glanders and farcy. There were the usual indications—discharge from the nostrils, ulceration of the membrane of those cavities, glandular swellings, and, in addition, a corded condition of the lymphatics along the course of the right saphena vein. It is remarked that, as the animal retained its vigor, it was allowed to remain in the stables until two other mares and a mule became infected, and exhibited symptoms of the same disease. The mare which was first attacked was killed, and on post-mortem examination the appearances left no room for doubt as to the nature of the disease. The three other animals were considered by M. Camoin to be favorable subjects for treatment, as they retained their strength and appearance of good health, in spite of the disease. Accordingly they were carefully isolated, well fed, and exercised every day. Each animal received every morning in a little honey one granule of salicylate of soda, ten granules of hyposulphite of strychnine, and fifteen granules of arsenious acid (arsenic).

The above described plan of treatment was continued for forty days, and during the time a little chlorine gas was discharged in the stable three times a week, the nostrils were frequently washed wtth carbolised water, and chloride of lime was sprinkled over the litter. The enlarged glands and farcy swelling were treated with a preparation containing bichloride of mercury. Under the treatment all the symptoms of glanders and farcy gradually disappeared, and the animals have remained in good health through the period of more than a year which has elapsed since the treatment was discontinued. M. Camoin remarks that the most scrupulous examination could not possibly reveal that these animals had ever been attacked by well-confirmed glanders.

M. Baumann's first experiment was made on a horse suffering from chronic glanders, which had been given up as incurable by the veterinary surgeon of the district. The animal was placed by itself in a large stable, and had at first five and then three granules of the sulphate or arseniate of strychnine every two hours, for about ten days. A solution of sulphate of zinc was injected into the nostrils, and the farcy boils were cauterised. The horse was so much better in ten days, that the medicine was reduced two granules at a time. The animal recovered, and has now been at work for a year.

M. Baumann considers that the arseniate of strychnine is the agent which produces the beneficial results, and looks upon the local remedies merely as useful adjuncts.

We may remark that we employed arseniate of strychnine some years ago in the treament of glanders in allopathic doses ; four grains daily were given in a ball with extract of gentian. The remedy certainly had a remarkable effect ; the ulcers of the nasal membrane quickly healed, and the discharge from the nostrils ceased ; the swollen glands also became diminished in size ; but, owing to the occurrence of tetanic spasms in the muscles of the back from the large doses of the agent, its use was discontinued, and after a time the symptoms of glanders returned.

On the general question of the curability of glanders, we may remark that, while the disease may be checked and relieved, perhaps cured so far as outward appearances are concerned, we should object to introduce such a "cured" horse into our stables ; and we doubt if any horseman of experience would be induced to accept as a gift the best hunter in the world, if he knew that the animal had suffered from "confirmed" glanders a year before. The only satisfactory way of dealing with the disease is by the policy of extermination.—*London Field.*

